# A Novel Strain of Fusarium oxysporum Virus 1 Isolated from *Fusarium oxysporum* f. sp. *niveum* Strain X-GS16 Influences Phenotypes of *F. oxysporum* Strain HB-TS-YT-1_hyg_

**DOI:** 10.3390/jof10040252

**Published:** 2024-03-27

**Authors:** Huihui Hua, Xinyi Zhang, Jie Xia, Xuehong Wu

**Affiliations:** College of Plant Protection, China Agricultural University, Haidian District, Beijing 100193, China; huahuihui202208@163.com (H.H.); zxy18511290531@163.com (X.Z.); xiajie202206@126.com (J.X.)

**Keywords:** *Fusarium oxysporum* f. sp. *niveum*, Fusarium oxysporum virus 1, hypovirulence, reduced sensitivity, difenoconazole, prochloraz, pydiflumetofen

## Abstract

A novel strain of Fusarium oxysporum virus 1 (FoV1) was identified from the *Fusarium oxysporum* f. sp. *niveum* strain X-GS16 and designated as Fusarium oxysporum virus 1-FON (FoV1-FON). The full genome of FoV1-FON is 2902 bp in length and contains two non-overlapping open reading frames (ORFs), ORF1 and ORF2, encoding a protein with an unknown function (containing a typical −1 slippery motif G_GAU_UUU at the 3′-end) and a putative RNA-dependent RNA polymerase (RdRp), respectively. BLASTx search against the National Center for the Biotechnology Information (NCBI) non-redundant database showed that FoV1-FON had the highest identity (97.46%) with FoV1. Phylogenetic analysis further confirmed that FoV1-FON clustered with FoV1 in the proposed genus Unirnavirus. FoV1-FON could vertically transmit via spores. Moreover, FoV1-FON was transmitted horizontally from the *F. oxysporum* f. sp. *niveum* strain X-GS16 to the *F. oxysporum* strain HB-TS-YT-1_hyg_. This resulted in the acquisition of the *F. oxysporum* strain HB-TS-YT-1_hyg_-V carrying FoV1-FON. No significant differences were observed in the sporulation and dry weight of mycelial biomass between HB-TS-YT-1_hyg_ and HB-TS-YT-1_hyg_-V. FoV1-FON infection significantly increased the mycelial growth of HB-TS-YT-1_hyg_, but decreased its virulence to potato tubers and sensitivity to difenoconazole, prochloraz, and pydiflumetofen. To our knowledge, this is the first report of hypovirulence and reduced sensitivity to difenoconazole, prochloraz, and pydiflumetofen in *F. oxysporum* due to FoV1-FON infection.

## 1. Introduction

Mycoviruses are widespread in all major taxa of fungi, including yeasts and mushrooms, as well as plant-, insect-, and human-pathogenic fungi, and other organisms such as oomycetes [1,2,3]. According to the latest taxonomy report of the International Committee on Virus Taxonomy (ICTV) and the accepted list of all mycoviruses in the ICTV VMR_MSL38_v2 (https://ictv.global/vmr, (accessed on 17 January 2024), there are 30 families and two genera containing a total of 491 species that infect fungi. The majority of them have an RNA genome, including the positive-sense single-stranded RNA (+ssRNA) genome, negative-sense single-stranded RNA (−ssRNA) genome, double-stranded RNA (dsRNA) genome, and single-stranded reverse-transcribing RNA (ssRNA-RT) genome; furthermore, there are several mycoviruses with a single-stranded DNA (ssDNA) genome [4].

Most mycoviruses produce latent infections and seldom induce symptoms [5,6,7]. However, some mycoviruses have been reported to cause dramatic changes in their hosts, including irregular growth and colony morphology, altered sexual or asexual reproduction, altered ecological fitness and virulence [7,8,9]. Furthermore, an increasing number of mycoviruses were identified to confer hypovirulence, such as the Fusarium graminearum virus 1 (FgV1) [10], Rosellinia necatrix megabirnavirus 1 (RnMBV1) [11], Heterobasidion partitivirus 13 (HetPV13) [12], Rhizoctonia solani partitivirus 2 strain BJ-1H (RsPV2-BJ-1H) [13], Stemphylium lycopersici alternavirus 1 (SlAV1) [14], and Fusarium avenaceum alternavirus 1 (FaAV1) [15].

*Fusarium oxysporum* is a globally dispersed soil-borne fungus, which has been positioned fifth among the top 10 economically significant phytopathogenic fungi [16]. To date, at least seventeen mycoviruses with the complete genomic sequence have been reported in *F. oxysporum*, including Fusarium oxysporum f. sp. dianthi mycovirus 1 (FodV1) [17,18,19], Fusarium oxysporum f. sp. dianthi mitovirus 1 (FodMV1) [20], Fusarium oxysporum f. sp. dianthi hypovirus 2 (FodHV2) [21], Fusarium oxysporum ourmia-like virus 1 (FoOuLV1) [22], Hadaka Virus 1 (HadV1) [23], Fusarium oxysporum alternavirus 1 (FoAV1) [24], Fusarium oxysporum mitovirus 1 (FoMV1) [25], Fusarium oxysporum mymonavirus 1 (FoMyV1) [26], as well as Fusarium oxysporum f. sp. cubense ourmia-like virus 1 (FocOLV1), FocOLV2, FocOLV3, FocOLV4, Fusarium oxysporum f. sp. cubense mitovirus 1 (FocMV1), FocMV2, FocMV4, Fusarium oxysporum f. sp. cubense mymonavirus 1 (FocMyV1), and Fusarium oxysporum f. sp. cubense negative-stranded RNA virus (FocNSRV1) [27]. Among these mycoviruses, FodV1, FoOuLV1, FoAV1, and FoMyV1 are capable of causing hypovirulence on their host fungi [18,22,25,26]. So far, no mycoviruses have been reported to be associated with *F. oxysporum* f. sp. *niveum*.

In the present study, a novel strain of Fusarium oxysporum virus 1 (FoV1) was isolated from the *F. oxysporum* f. sp. *niveum* strain X-GS16 and tentatively designated as Fusarium oxysporum virus 1-FON (FoV1-FON), which was the causal agent of watermelon Fusarium wilt. An assay on the vertical transmission of FoV1-FON via spores was conducted. The horizontal transmission of FoV1-FON from the *F. oxysporum* f. sp. *niveum* strain X-GS16 to the *F. oxysporum* strain HB-TS-YT-1_hyg_, causing potato dry rot through hyphal contact using pairing cultures, was carried out. In addition, the impacts on the colony growth, sporulation, virulence, and sensitivity to three fungicides (difenoconazole, prochloraz, and pydiflumetofen) of the *F. oxysporum* strain HB-TS-YT-1_hyg_ induced by FoV1-FON were studied.

## 2. Materials and Methods

### 2.1. Fungal Strains

Three strains (*F. oxysporum* f. sp. *niveum* strain X-GS16, *F. oxysporum* strain HB-TS-YT-1_hyg_, and *F. oxysporum* strain HB-TS-YT-1_hyg_-V) were used in this study. X-GS16 was isolated from diseased watermelon roots with the symptom of Fusarium wilt and identified to be *F. oxysporum* f. sp. *niveum*, according to the methods described previously [28,29,30], which was collected from Tianshui city, Gansu province of China. HB-TS-YT-1_hyg_ conferring a hygromycin resistance was confirmed to be FoV1-FON-free based on the reverse transcription-polymerase chain reaction (RT-PCR) detection of FoV1-FON using FoV1-FON-specific primers (FoV1-FON-F and FoV1-FON-R listed in Table 1), which was obtained by transforming the hygromycin B phosphotransferase gene to the *F. oxysporum* strain HB-TS-YT-1 (the causal agent of potato dry rot) successfully in our previous study [15]. HB-TS-YT-1_hyg_-V carrying FoV1-FON was obtained through hyphal contact by pairing cultures of the colonies from *F. oxysporum* f. sp. *niveum* strain X-GS16 (donor strain) and *F. oxysporum* strain HB-TS-YT-1_hyg_ (recipient strain). All the three strains were grown on PDA plates for 7 d at 25 °C in the dark for subsequent use.

### 2.2. Extraction and Purification of RNA

The dsRNA was extracted using the CF-11 cellulose (Sigma-Aldrich, St. Louis, MI, USA) chromatography method [31], then purified with a gel extraction kit (Aidlab Biotechnologies, Beijing, China) after being treated with DNase I (TaKaRa, Dalian, China) and S1 Nuclease (TaKaRa, Dalian, China) [15]. TRIpure Reagent (Aidlab Biotechnologies) was used for the total RNA extraction according to the manufacturer’s instructions.

### 2.3. Synthesis and Molecular Cloning of Complementary DNA (cDNA)

The synthesis and molecular cloning of cDNA were performed as previously described [32]. Briefly, the purified dsRNA was coupled with a tagged random primer (RACE3RT: 5′-CGATCGATCATGATGCAATGCNNNNNN-3′) for reverse transcription, and the obtained random cDNA products were amplified using a single specific primer (RACE3: 5′-CGATCGATCATGATGCAATGC-3′). Then, the resulting DNA segments were purified (TaKaRa, Dalian, China) and cloned into the pTOPO-TA vector (Aidlab Biotechnologies, Beijing, China). After transforming into *Trelif*™ 5α chemically competent cells (Tsingke Biotech Co., Ltd. Beijing, China), positive clones with inserts of >500 bp in length were sent to Tsingke Biotech Co., Ltd. for sequencing. Final sequences were determined using at least three clones, and assembled using DNAMAN 7.0 (Lynnon Biosoft, CA, USA). Gaps between non-overlapping cDNA clones were determined using RT-PCR amplification with specific primers, FoV1-FON-GAP1-F and FoV1-FON-GAP1-R, which were listed in Table 1.

The 5′- and 3′-terminal sequences were obtained using an RNA-ligase-mediated rapid amplification of cDNA ends (RLM-RACE) method, as described previously [33]. Sequencing was conducted by Tsingke Biotech Co., Ltd. Finally, the whole genome sequence was assembled using DNAMAN 7.0 (Lynnon Biosoft, CA, USA).

### 2.4. Sequence Analysis, Alignment, and Phylogenetic Analysis

Putative open reading frames (ORFs) in dsRNA were identified using the ORF Finder program on the NCBI website (https://www.ncbi.nlm.nih.gov/, (accessed on 17 January 2024) with the standard genetic code. The Conserved Domain Database (https://www.ncbi.nlm.nih.gov/Structure/cdd/wrpsb.cgi, (accessed on 17 January 2024) was utilized to identify conserved motifs. CLUSTAL_X 1.8 was used to conduct multiple alignment [34]. A phylogenetic tree was constructed based on the deduced amino acid (aa) sequence of RNA-dependent RNA polymerase (RdRp) using the maximum-likelihood (ML) method in MEGA version 6.0 software with a bootstrap value of 1000 [34]. Hypothetical RNA secondary structures of 3′-terminal sequence of dsRNA were generated using the RNAfold webserver (http://rna.tbi.univie.ac.at/cgi-bin/RNAWebSuite/RNAfold.cgi, (accessed on 17 January 2024).

### 2.5. Elimination of FoV1-FON from X-GS16

According to the methods described previously [2,13,15,35,36,37,38], we tried five methods, namely, single-spore isolation, hyphal tipping, protoplast regeneration, cycloheximide treatment, and ribavirin treatment, to remove FoV1-FON from X-GS16.

The collected mycelia of the strains derived from X-GS16 were subjected to the extraction of total RNA, which was used as a template to conduct RT-PCR using FoV1-FON-specific primers (FoV1-FON-F and FoV1-FON-R listed in Table 1). Gel electrophoretic profiles of RT-PCR products were performed to confirm whether FoV1-FON was eliminated from X-GS16 successfully [15,36].

### 2.6. Vertical and Horizontal Transmission of FoV1-FON

The vertical transmission of FoV1-FON via spores was assessed using the single-spore isolation method mentioned above [35,36].

The horizontal transmission of FoV1-FON through hyphal contact using pairing cultures was evaluated according to the methods described previously [15,36,39]. ZnCl_2_-amended PDA plates (1.5 mM) were used to overcome mycelial incompatibility between the donor strain (X-GS16) and recipient strain (HB-TS-YT-1_hyg_). The mycelia of each derivative strain were collected to extract total RNA. The extracted total RNA was used as a template to conduct RT-PCR using FoV1-FON-specific primers (FoV1-FON-F and FoV1-FON-R listed in Table 1). Gel electrophoretic profiles of RT-PCR products were performed to confirm the positive presence of total RNA in the derivative strains, and, thus, determined whether FoV1-FON had been horizontally transmitted from X-GS16 to HB-TS-YT-1_hyg_ or not. In addition, gel electrophoretic profiles of the dsRNA of derivative strains were carried out to further confirm the existence of FoV1-FON in HB-TS-YT-1_hyg_. The derivative strain verified to carry FoV1-FON was designated as HB-TS-YT-1_hyg_-V.

### 2.7. Effect of FoV1-FON on Phenotypes of F. oxysporum Strain HB-TS-YT-1_hyg_

The effect of FoV1-FON on the colony growth, sporulation, and mycelial biomass of HB-TS-YT-1_hyg_ and HB-TS-YT-1_hyg_-V was determined as described in our previously studies [13,15,36]. The mycelial biomass was evaluated by the dry weight of the mycelia collected 7 d after incubation. Three biological replicates and three technical replicates were applied in the experiment. The statistical differences were determined by a *t*-test through the Graphpad Prism software (version 9.5).

### 2.8. Pathogenicity Test of HB-TS-YT-1_hyg_ and HB-TS-YT-1_hyg_-V

A pathogenicity test of the two strains, HB-TS-YT-1_hyg_ and HB-TS-YT-1_hyg_-V, was conducted as the method described previously [15,40]. Three biological replicates and three technical replicates were applied in the experiment. The statistical differences were determined by a *t*-test through the Graphpad Prism software (version 9.5).

### 2.9. Sensitivity of HB-TS-YT-1_hyg_ and HB-TS-YT-1_hyg_-V to Difenoconazole, Prochloraz, and Pydiflumetofen

The sensitivity of the two strains, HB-TS-YT-1_hyg_ and HB-TS-YT-1_hyg_-V, to difenoconazole [Active ingredient (AI): 94.50%], prochloraz (AI: 98.00%), and pydiflumetofen (AI: 98.00%) was evaluated in vitro, as described in a previous study [41]. The PDA media were amended with difenoconazole, prochloraz, and pydiflumetofen to establish the final concentrations of 5.00, 1.00, 0.50, 0.10, and 0.05 µg/mL, 0.50, 0.10, 0.05, 0.01, and 0.005 µg/mL, and 1.00, 0.50, 0.10, 0.05, and 0.01 µg/mL, respectively. The median effective concentration (EC_50_) of difenoconazole, prochloraz, and pydiflumetofen against the two strains was calculated as described by Zhao et al. [41]. Four replicates were used for each strain–fungicide combination, and the experiment was repeated three times. The *t*-test was performed using Graphpad Prism version 9.5 for the statistical analysis.

## 3. Results

### 3.1. Complete Sequence and Phylogenetic Analysis of FoV1-FON

**Figure 1:**
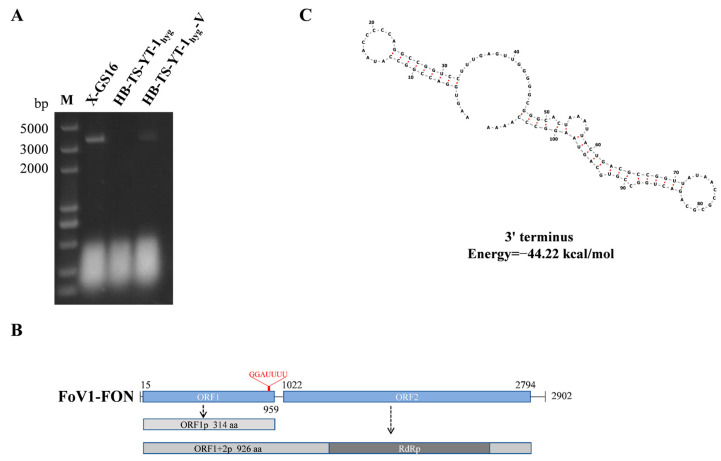
Characterization of Fusarium oxysporum virus 1-FON (FoV1-FON) isolated from the *Fusarium oxysporum* f. sp. *niveum* strain X-GS16. (**A**) Gel electrophoretic profiles of the double-stranded RNA (dsRNA) extracted from X-GS16, HB-TS-YT-1_hyg_, and HB-TS-YT-1_hyg_-V, which was treated with DNase I and S1 Nuclease; Lane M: DNA molecular marker DL 5000. (**B**) Schematic diagram of the genomic organization of FoV1-FON. Open reading frames (ORFs) and untranslated regions (UTRs) are indicated by rectangles and single lines, respectively. (**C**) Predicted secondary structure of the 3′- UTR of FoV1-FON. The red color means complementary base pairing.

**Figure 2:**
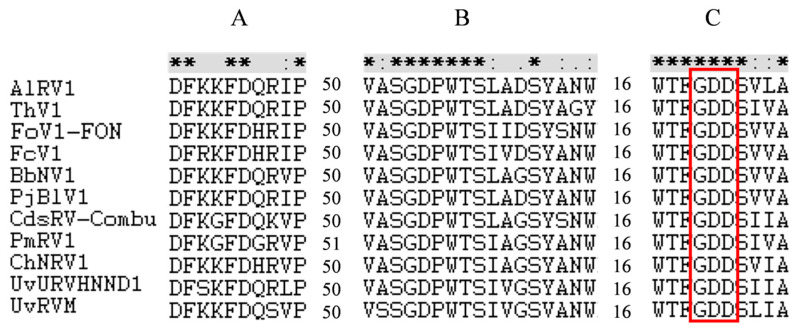
Multiple alignment of the amino acid (aa) sequence of RdRp of Fusarium oxysporum virus 1-FON (FoV1-FON) and ten reference viruses of the genus Unirnavirus. Three conserved motifs (motif A, motif B, and motif C) were identified to be present in FoV1-FON and the 10 representative members of the genus Unirnavirus. Asterisks represent identical amino acid residues, colons represent amino acids with a high level of chemical similarity, and dots represent amino acid residues with a low level of chemical similarity. The red box indicates the highly conserved GDD tripeptide. The names and GenBank accession numbers of these ten representative members were listed in Table S1.

The electrophoresis of the *F. oxysporum* f. sp. *niveum* strain X-GS16 dsRNA extracts resulted in an approximately 3 kb single band (Figure 1A). The complete nucleotide sequence of the dsRNA was determined to be 2902 nt in length with a G+C content of 53.4%. The genome sequence was deposited in GenBank under the accession number PP404034. BLASTx search against National Center for the Biotechnology Information (NCBI) non-redundant database (accessed on 17 January 2024) showed that it was the most closely related to Fusarium oxysporum virus 1 (FoV1) (identity 97.46%; E value 0; query cover 60%), and designated as FoV1-FON. The genome of FoV1-FON harbors two discontinuous ORFs (ORF1 and ORF2) on the positive strand (Figure 1B). ORF1 encodes an unknown function protein of 314 aa with an estimated mass of 34.27 kDa. The typical −1 slippery motif G_GAU_UUU [42,43] was identified immediately before the stop codon UAA of ORF1 (between 950 nt and 956 nt), suggesting that a programmed ribosomal frameshifting (PRF) was utilized for ORF2 translation. In the case of PRF, the hypothetical fusion protein ORF1+2p contains 926 aa, with an estimated mass of 104.18 kDa. ORF2 sequence contains a predicted RdRp domain, and the palm motifs A, B, and C (Figure 2) were identified within the RdRp domain of FoV1-FON and ten reference Unirnaviruses, whose full name and GenBank accession number were listed in Table S1.

The untranslated regions (UTRs) flanking the ORF at the 5′- and 3′-end were detected to be 14 nt and 108 nt, respectively. Interestingly, stable stem-loop structures were predicted to present in the 3′-UTR of FoV1-FON (Figure 1C, with minimum optimal energy ∆G= −44.22 kcal/mol). This prediction was also evidenced in 3′-UTR of another Unirnavirus, Fusarium culmorum virus 1 (FcV1) [43]. Stem loop structures were common in viral UTRs, and might be biologically important in replication and translation [43].

**Figure 3:**
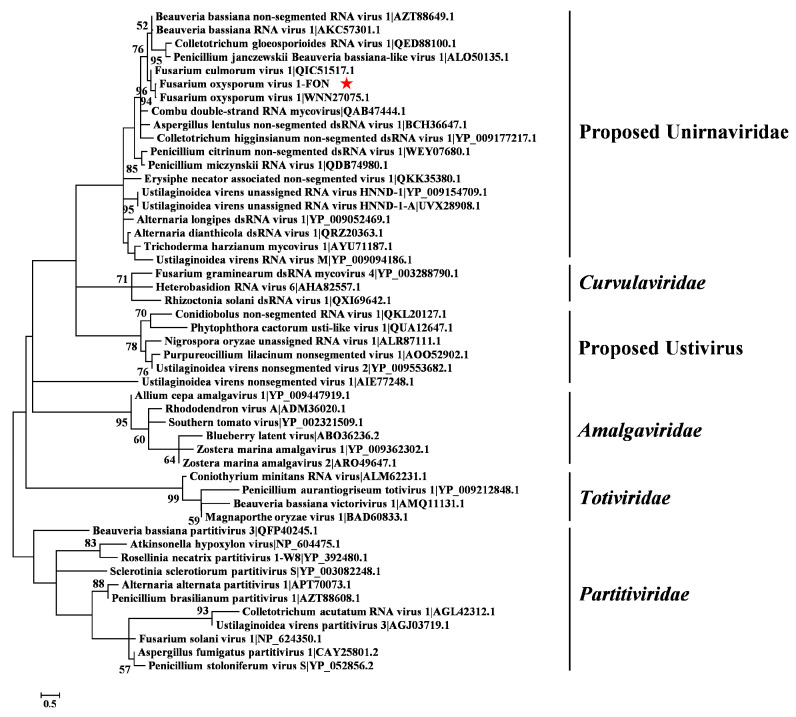
Phylogenetic tree constructed based on amino acid (aa) sequence of RNA-dependent RNA polymerase (RdRp) of Fusarium oxysporum virus 1-FON (FoV1-FON) and 48 representative members in the five families (*Amalgaviridae*, *Curvulaviridae*, *Partitiviridae*, *Totiviridae*, and the proposed family Unirnaviridae) and the proposed genus Ustivirus using the maximum-likelihood (ML) method with 1000 bootstrap replicates. The bar scale represents a genetic distance of 0.5 aa substitutions per site. Bootstrap values <50% are hidden. The red star represents the position of FoV1-FON. The name and GenBank accession number of these 48 representative members were listed in Table S2.

A maximum-likelihood phylogenetic analysis based on the aa sequence of the RdRp of FoV1-FON and 48 representative members in the five families (*Amalgaviridae*, *Curvulaviridae*, *Partitiviridae*, *Totiviridae*, and the proposed family Unirnaviridae) and the proposed genus Ustivirus revealed that FoV1-FON clustered together with members of the proposed family Unirnaviridae and was the most closely related to FoV1 (Figure 3), whose full name and GenBank accession number were listed in Table S2. Collectively, all the results indicated that FoV1-FON is a novel strain of FoV1 belonging to the genus Unirnavirus within the proposed family Unirnaviridae.

### 3.2. Vertical and Horizontal Transmission of FoV1-FON

**Figure 4:**
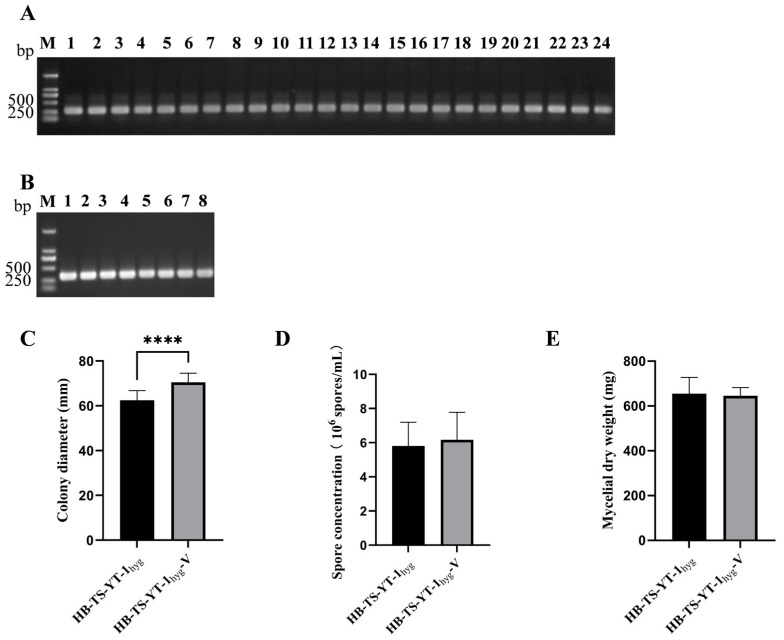
Vertical and horizontal transmission of Fusarium oxysporum virus 1-FON (FoV1-FON), and the effect of FoV1-FON on the phenotypes of *F. oxysporum* strain HB-TS-YT-1_hyg_. (**A**) Confirmation of the presence of FoV1-FON in the 24 single-spore cultures derived from X-GS16 by 1.0% (*w*/*v*) agarose gel electrophoretic profiles of reverse transcription-polymerase chain reaction (RT-PCR) products. Lane M: DNA molecular marker DL 2000; Lane 1–24: Products of RT-PCR using viral-specific primers (Table 1) designed based on the nucleotide sequence of FoV1-FON. (**B**) Confirmation of the presence of FoV1-FON in HB-TS-YT-1_hyg_-V derived from HB-TS-YT-1_hyg_ by 1.0% (*w*/*v*) agarose gel electrophoretic profiles of RT-PCR products. Lane M: DNA molecular marker DL 2000; Lane 1–8: Products of RT-PCR using viral-specific primers (Table 1) designed based on the nucleotide sequence of FoV1-FON. (**C**) Colony diameter of the two strains, HB-TS-YT-1_hyg_ and HB-TS-YT-1_hyg_-V, cultured on potato dextrose agar (PDA) plates at 25 °C in darkness for 6 d. (**D**) Spores produced by the two strains, HB-TS-YT-1_hyg_ and HB-TS-YT-1_hyg_-V, cultured on PDA plates at 25 °C for 7 d in the dark. (**E**) Dry weight of mycelial biomass generated by the two strains, HB-TS-YT-1_hyg_ and HB-TS-YT-1_hyg_-V, cultured in potato dextrose broth (PDB) for 7 d at 25 °C in the dark. Asterisks indicate different levels of significant difference (**** *p* < 0.0001) between HB-TS-YT-1_hyg_ and HB-TS-YT-1_hyg_-V as determined by the *t*-test using GraphPad Prism version 9.5 software.

Gel electrophoretic profiles of RT-PCR products from 24 single-spore cultures derived from X-GS16 were evaluated, and the results indicated that FoV1-FON could vertically transmit via spores (Figure 4A).

The attempts to eliminate FoV1-FON from X-GS16 failed. The *F. oxysporum* strain HB-TS-YT-1_hyg_-V carrying FoV1-FON was obtained successfully by pairing cultures and verified by gel electrophoretic profiles of the detection of FoV1-FON using RT-PCR (Figure 4B) with the specific primers (FoV1-FON-F and FoV1-FON-R listed in Table 1) and dsRNA of HB-TS-YT-1_hyg_-V (Figure 1A).

### 3.3. Effect of FoV1-FON on Phenotypes of F. oxysporum Strain HB-TS-YT-1_hyg_

The average colony growth rate (10.92 mm/d) of HB-TS-YT-1_hyg_-V was significantly higher than that (9.58 mm/d) of HB-TS-YT-1_hyg_ (Figure 4C). However, no significant difference in the average spore concentration was observed between HB-TS-YT-1_hyg_ (5.81 × 10^6^ spores/mL) and HB-TS-YT-1_hyg_-V (6.17 × 10^6^ spores/mL) (Figure 4D). Moreover, the average dry weight of mycelial biomass (655.0 mg) of HB-TS-YT-1_hyg_ was similar to that (645.3 mg) of HB-TS-YT-1_hyg_-V (Figure 4E).

### 3.4. Effect of FoV1-FON on Virulence of F. oxysporum Strain HB-TS-YT-1_hyg_

**Figure 5:**
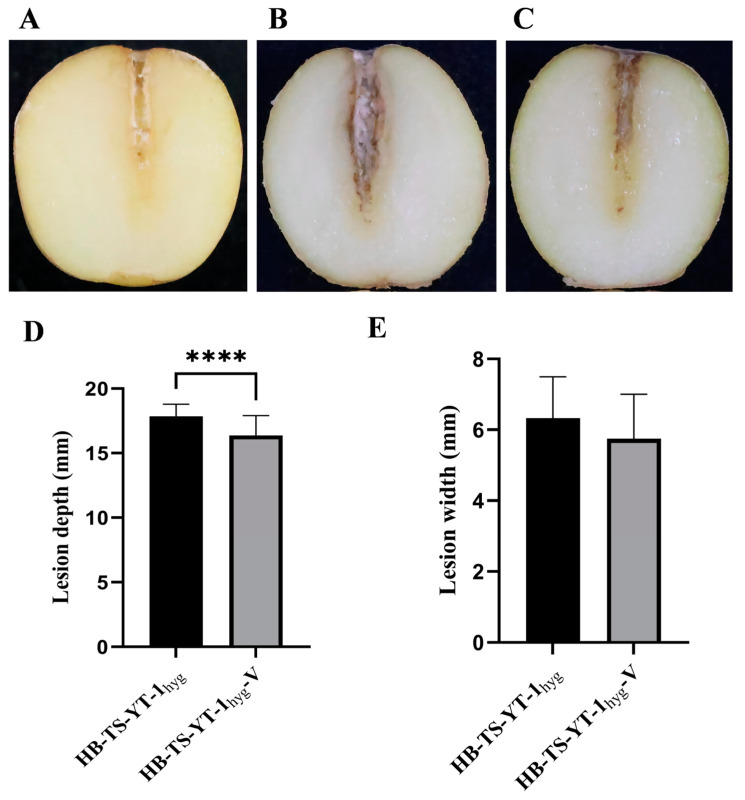
Virulence of the two strains of *Fusarium oxysporum*, HB-TS-YT-1_hyg_ and HB-TS-YT-1_hyg_-V, to potato tubers. (**A**) Potato tubers inoculated with sterile distilled water. (**B**) Potato tubers inoculated with spore suspension of HB-TS-YT-1_hyg_. (**C**) Potato tubers inoculated with spore suspension of HB-TS-YT-1_hyg_-V. (**D**) Depth of lesions on potato tubers infected by the two strains, HB-TS-YT-1_hyg_ and HB-TS-YT-1_hyg_-V. (**E**) Width of lesions on potato tubers infected by the two strains, HB-TS-YT-1_hyg_ and HB-TS-YT-1_hyg_-V. Asterisks indicate different levels of significant difference (**** *p* < 0.0001) between HB-TS-YT-1_hyg_ and HB-TS-YT-1_hyg_-V as determined by the *t*-test using GraphPad Prism version 9.5 software.

The two strains, HB-TS-YT-1_hyg_ and HB-TS-YT-1_hyg_-V, all formed a white colony on the wounded surface of potato tubers (Figure 5A–C). The average depth (17.9 mm) of the lesions on potato tubers caused by HB-TS-YT-1_hyg_ was significantly greater than that (16.4 mm) of the lesions on potato tubers caused by HB-TS-YT-1_hyg_-V (Figure 5D). However, no significant difference was found on the average width of the lesions on potato tubers incited by HB-TS-YT-1_hyg_ and HB-TS-YT-1_hyg_-V, which was 6.3 mm and 5.8 mm, respectively (Figure 5E). These results showed that FoV1-FON infection conferred hypovirulence on the *F. oxysporum* strain HB-TS-YT-1_hyg_.

### 3.5. Effect of FoV1-FON on the Sensitivity of F. oxysporum Strain HB-TS-YT-1_hyg_ to Difenoconazole, Prochloraz, and Pydiflumetofen

**Figure 6:**
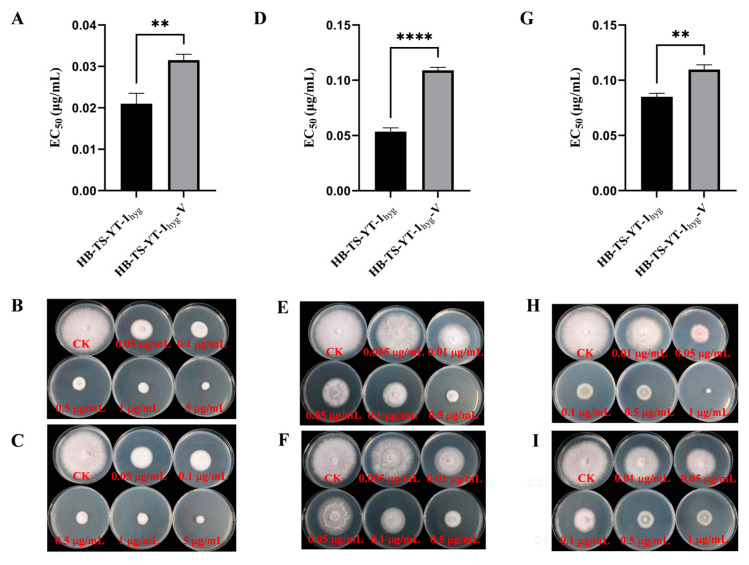
Sensitivity of the two strains of *Fusarium oxysporum*, HB-TS-YT-1_hyg_ and HB-TS-YT-1_hyg_-V, to difenoconazole, prochloraz, and pydiflumetofen. (**A**) Median effective concentration (EC_50_) of difenoconazole against the two strains, HB-TS-YT-1_hyg_ and HB-TS-YT-1_hyg_-V. (**B**) Effect of difenoconazole on the colony growth of HB-TS-YT-1_hyg_. (**C**) Effect of difenoconazole on the colony growth of HB-TS-YT-1_hyg_-V. (**D**) EC_50_ of prochloraz against the two strains, HB-TS-YT-1_hyg_ and HB-TS-YT-1_hyg_-V. (**E**) Effect of prochloraz on the colony growth of HB-TS-YT-1_hyg_. (**F**) Effect of prochloraz on the colony growth of HB-TS-YT-1_hyg_-V. (**G**) EC_50_ of pydiflumetofen against the two strains, HB-TS-YT-1_hyg_ and HB-TS-YT-1_hyg_-V. (**H**) Effect of pydiflumetofen on the colony growth of HB-TS-YT-1_hyg_. (**I**) Effect of pydiflumetofen on the colony growth of HB-TS-YT-1_hyg_-V. Asterisks indicate different levels of significant difference (** *p* < 0.01; **** *p* < 0.0001) between HB-TS-YT-1_hyg_ and HB-TS-YT-1_hyg_-V as determined by the *t*-test using GraphPad Prism version 9.5 software.

The sensitivity of the two strains, HB-TS-YT-1_hyg_ and HB-TS-YT-1_hyg_-V, to difenoconazole, prochloraz, and pydiflumetofen was studied in vitro. The results indicated that these three fungicides could effectively inhibit the colony growth of the two strains (Figure 6). The EC_50_ value of difenoconazole (0.0315 g/mL), prochloraz (0.1090 g/mL), and pydiflumetofen (0.1097 g/mL) against HB-TS-YT-1_hyg_-V was significantly higher than that of difenoconazole (0.0210 g/mL), prochloraz (0.0536 g/mL), and pydiflumetofen (0.0851 g/mL) against HB-TS-YT-1_hyg_, respectively (Figure 6), indicating that FoV1-FON infection was related to a decreased sensitivity of the *F. oxysporum* strain HB-TS-YT-1_hyg_ to difenoconazole, prochloraz, and pydiflumetofen.

## 4. Discussion

In the present study, we identified and characterized a novel strain of FoV1 associated with the *F. oxysporum* f. sp. *niveum* strain X-GS16 causing watermelon Fusarium wilt, which was designated as FoV1-FON. FoV1-FON could vertically transmit efficiently via spores, and horizontally transmit from X-GS16 to HB-TS-YT-1_hyg_. No significant differences were observed in sporulation and mycelial biomass between HB-TS-YT-1_hyg_ and HB-TS-YT-1_hyg_-V. FoV1-FON significantly increased the mycelial growth of HB-TS-YT-1_hyg_-V but decreased its virulence to potato tubers and sensitivity to difenoconazole, prochloraz, and pydiflumetofen. To our knowledge, this is the first report of an Unirnavirus affecting the virulence and sensitivity to fungicides of *F. oxysporum*.

The proposed genus Unirnavirus and the proposed family Unirnaviridae were suggested to be established in 2015 [44] and in 2017 [45], respectively. Later, several mycoviruses discovered in different fungal species also supported the establishment of Unirnaviridae, such as FcV1 infecting *F. culmorum* [43] and Penicillium citrinum non-segmented dsRNA virus 1 infecting *Penicillium citrinum* [46]. In previous studies, it was reported that the members of the proposed family Unirnaviridae were related to monopartite viruses with a similar genome organization; these belong to the family *Amalgaviridae* or the proposed genus Ustivirus [42,43,46]. In this study, phylogenetic analysis showed that FoV1-FON formed a well-supported independent clade together with members of the genus Unirnavirus within Unirnaviridae, and presented a distant relation with amalgaviruses or ustiviruses. Therefore, the results of our study also supported that Unirnaviridae should be established as a new family, thus broadening the diversity of the mycoviruses belonging to this taxon.

The function of the protein encoded by ORF1 in Unirnaviruses is currently not clear. Conserved α-helical coiled-coil structures were predicted in the proteins encoded by ORF1, which might play a vital role in Unirnaviruses [43]. Campo et al. [47] predicted that the putative capsid protein encoded by the ORF1 of Colletotrichum higginsianum non-segmented dsRNA virus 1 (ChNRV1) was present at very low amounts in the *Colletotrichum higginsianum* wild-type strain; however, repeated attempts to observe ChNRV1 virions using electron microscopy were unsuccessful. In the present study, the attempt to extract and purify virus particles from X-GS16 also failed.

The classical slippery sequence for −1 PRF is X_XXY_YYZ, where XXX is any three of the same nucleotides, although several deviations such as GGA are tolerated; YYY is AAA or UUU (do not confuse with the use of Y for pyrimidine in other cases); Z is A, C, or U [42]. The nucleotides located immediately or soon before the stop codon in ORF1 were observed in the Unirnaviruses reported previously. Among them, Penicillium miczynskii RNA virus 1 (PmRV1) and Ustilaginoidea virens unassigned RNA virus HNND-1 (UvURV-HNND1) contain the U_UUA_AAC heptamer, while the remaining Unirnaviruses have a −1 PRF slippery motif consisting of G_GAU_UUZ (Z stands for A, C or U) (Table S3). As described by Mahillon et al. [43], the −1 PRF slippery motif immediately or soon before the stop codon of ORF1 is one of the features of Unirnaviruses. Similar to ChNRV1, FcV1 was believed to produce a fusion protein due to the −1 PRF, which is likely to be associated with viral replication [43,47]. In the current study, the typical −1 slippery motif G_GAU_UUU was present at the 3′-end of ORF1 of FoV1-FON, further supporting that FoV1-FON was a new strain of FoV1, an Unirnavirus within the proposed family Unirnaviridae.

Virus-infected and virus-free isogenic lines are required for an effective investigation of the impact of a virus on its host, and the common method is to eliminate the virus from infected strains or introduce the virus into virus-free fungal isolates [48]. In this study, we used five methods (single-spore isolation, hyphal tipping, protoplast regeneration, cycloheximide treatment, and ribavirin treatment) to eliminate FoV1-FON from X-GS16, but we failed. In the previous study, FcV1 was not eliminated from the *F. culmorum* strain A104-1 successfully [43]. It seemed that Unirnaviruses had evolved efficient strategies to be vertically retained in their respective hosts. Fortunately, FoV1-FON was successfully horizontally transmitted from X-GS16 to HB-TS-YT-1_hyg_ by hyphal contact, and, thus, HB-TS-YT-1_hyg_-V carrying FoV1-FON was obtained, which made it possible to study the effect of FoV1-FON on the phenotypes of its host fungus.

When one *Monilinia fructicola* isolate, which is a fundamental agent causing brown rot on *Prunus* species, was co-infected by three mycoviruses, its mycelial growth rate increased, but there were no visible effects on lesions growth patterns on plums [49]. FoMyV1 infection reduced vegetative growth and spore production of its fungal host, *F. oxysporum*, but did not alter its virulence [26]. RsPV2-BJ-1H infected *Rhizoctonia solani* and did not significantly affect the hyphal width, sclerotia dry weight, and radial growth rate of its host fungus, but significantly decreased the virulence of its host [13]. FaAV1 could not alter the colony morphology and spore production of its fungal host, but increased the mycelial biomass and virulence of its host fungus [15]. In this study, FoV1-FON could reduce the virulence of *F. oxysporum* to potato tubers and increase the mycelial growth rate of its host fungus; however, no significant differences were observed between HB-TS-YT-1_hyg_ and HB-TS-YT-1_hyg_-V in terms of spore production and the dry weight of the mycelial biomass.

Difenoconazole, a demethylation inhibitor (DMI) fungicide, has been widely utilized to control black spot disease caused by *Alternaria* [50,51], as well as Fusarium wilt incited by *F. oxysporum* [52,53]. Prochloraz is also a DMI fungicide, which is used to manage spot disease caused by *A. tenuissima* [54] and the Fusarium head blight incited by *F. graminearum* [55] or Fusarium wilt induced by *F. oxysporum* [53]. Pydiflumetofen, as a succinate dehydrogenase inhibitor (SDHI) fungicide, can effectively control Alternaria leaf spot caused by *A. alternata* [56] and Fusarium wilt incited by *F. oxysporum* f. sp. *niveum* [57]. In our previous studies, the infection of Alternaria alternata chrysovirus 1-AT1 (AaCV1-AT1) could reduce the sensitivity of its host fungus, *A. tenuissima*, to difenoconazole [58]; Alternaria alternata botybirnavirus 1-AT1 (AaBRV1-AT1) also decreased the sensitivity of its host fungus, *A. tenuissima*, to difenoconazole [36]. Similarly, in the present study, FoV1-FON reduced the sensitivity of *F. oxysporum* to difenoconazole; moreover, FoV1-FON lowered the sensitivity of *F. oxysporum* to prochloraz and pydiflumetofen. This is the first report of a mycovirus affecting the sensitivity of its host fungus to pydiflumetofen.

## Figures and Tables

**Table 1:** Primers used to determine the complete genome sequence of the Fusarium oxysporum virus 1-FON (FoV1-FON) in this study.

Primer Name	Sequence (5′-3′)
PC3-T7 Loop adapter	p-GGATCCCGGGAATTCGGTAATACGACTCACTATA TTTTTATAGTGAGTCGTATTA-OH
PC2	CCGAATTCCCGGGATCC
FoV1-FON-GAP1-F	AAAAAGAGTAGCACTGGAACGAGA
FoV1-FON-GAP1-R	CAGGAAATACAGGGAGAGAAAAGA
FoV1-FON-F	GGCTTACCTCACCTTCTTTTAC
FoV1-FON-R	TGTTGCCAGACACATCCTTATC
FoV1-FON-5end-1	GACGTGACTTATACTCCTGAGCGACCT
FoV1-FON-5end-2	AAACGATCTCCACGGGTGACAGC
FoV1-FON-3end-1	GAGGTTCGTCTTAGAAGCCTACTGGG
FoV1-FON-3end-2	TGGTTCTTTTCTCTCCCTGTATTTC

## Data Availability

The sequence of the Fusarium oxysporum virus 1-FON (FoV1-FON) reported in the present manuscript has been deposited in the GenBank database under accession number PP404034.

## Supplementary Materials

The following supporting information can be downloaded at: https://www.mdpi.com/article/10.3390/jof10040252/s1, Table S1: The information of ten reference unirnaviruses retrieved from GenBank database (National Center for the Biotechnology Information) and used to conduct multiple alignment. Table S2: The information of 48 representative members in the five families (*Amalgaviridae*, *Curvulaviridae*, *Partitiviridae*, *Totiviridae*, and the proposed family Unirnaviridae) and the proposed genus Ustivirus retrieved from GenBank database (National Center for the Biotechnology Information) and used to conduct phylogenetic analysis. Table S3: The information of 18 representative members in the proposed family Unirnaviridae retrieved from GenBank database (National Center for the Biotechnology Information) and used to analyze the hypothetical −1 frameshifting motifs.
