# Supplementary material for: A Novel Strain of Fusarium oxysporum Virus 1 Isolated from Fusarium oxysporum f. sp. niveum Strain X-GS16 Influences Phenotypes of F. oxysporum Strain HB-TS-YT-1hyg
Source: J Fungi (Basel). 2024 Mar 27;10(4):252. doi: 10.3390/jof10040252 (PMC11050907; doi:10.3390/jof10040252)
Supplement: Supplementary file 1 [file jof-10-00252-s001.zip › Table S1.docx]

**Table S1.** The information of ten reference unirnaviruses retrieved from GenBank database (National Center for the Biotechnology Information) and used to conduct multiple alignment.

| **Reference Virus** | **GenBank Accession Number** |
| --- | --- |
| Alternaria longipes dsRNA virus 1 (AlRV1) | YP_009052469.1 |
| Trichoderma harzianum mycovirus 1 (ThV1) | AYU71187.1 |
| Fusarium culmorum virus 1 (FcV1) | QIC51517.1 |
| Beauveria bassiana non-segmented RNA virus 1 (BbNRV1) | AZT88649.1 |
| Penicillium janczewskii Beauveria bassiana-like virus 1 (PjBlV1) | ALO50135.1 |
| Combu double-strand RNA mycovirus (CdsRV1) | QAB47444.1 |
| Penicillium miczynskii RNA virus 1 (PmRV1) | QDB74980.1 |
| Colletotrichum higginsianum non-segmented dsRNA virus 1 (ChNRV1) | YP_009177217.1 |
| Ustilaginoidea virens unassigned RNA virus HNND-1 (UvURV-HNND1) | YP_009154709.1 |
| Ustilaginoidea virens RNA virus M (UvRVM) | YP_009094186.1 |
