# Supplementary material for: A Novel Strain of Fusarium oxysporum Virus 1 Isolated from Fusarium oxysporum f. sp. niveum Strain X-GS16 Influences Phenotypes of F. oxysporum Strain HB-TS-YT-1hyg
Source: J Fungi (Basel). 2024 Mar 27;10(4):252. doi: 10.3390/jof10040252 (PMC11050907; doi:10.3390/jof10040252)
Supplement: Supplementary file 1 [file jof-10-00252-s001.zip › Table S2.docx]

**Table S2.** The information of 48 representative members in the five families (*Amalgaviridae*, *Curvulaviridae*, *Partitiviridae*, *Totiviridae*, and the proposed family Unirnaviridae) and the proposed genus Ustivirus retrieved from GenBank database (National Center for the Biotechnology Information) and used to conduct phylogenetic analysis.

| **Reference Virus** | **Family or Genus** | **GenBank Accession Number** |
| --- | --- | --- |
| Allium cepa amalgavirus 1 (AcAV1) | *Amalgaviridae* | YP_009447919.1 |
| Blueberry latent virus (BlV) | *Amalgaviridae* | ABO36236.2 |
| Rhododendron virus A (RVA) | *Amalgaviridae* | ADM36020.1 |
| Southern tomato virus (StV) | *Amalgaviridae* | YP_002321509.1 |
| Zostera marina amalgavirus 1 (ZmAV1) | *Amalgaviridae* | YP_009362302.1 |
| Zostera marina amalgavirus 2 (ZmAV2) | *Amalgaviridae* | ARO49647.1 |
| Fusarium graminearum dsRNA mycovirus 4 (FgV4) | *Curvulaviridae* | YP_003288790.1 |
| Heterobasidion RNA virus 6 (HRV6) | *Curvulaviridae* | AHA82557.1 |
| Rhizoctonia solani dsRNA virus 1 (RsRV1) | *Curvulaviridae* | QXI69642.1 |
| Alternaria alternata partitivirus 1 (AaPV1) | *Partitiviridae* | APT70073.1 |
| Aspergillus fumigatus partitivirus 1 (AfPV1) | *Partitiviridae* | CAY25801.2 |
| Atkinsonella hypoxylon virus (AhV) | *Partitiviridae* | NP_604475.1 |
| Beauveria bassiana partitivirus 3 (BbPV3) | *Partitiviridae* | QFP40245.1 |
| Colletotrichum acutatum RNA virus 1 (CaRV1) | *Partitiviridae* | AGL42312.1 |
| Fusarium solani virus 1 (FsV1) | *Partitiviridae* | NP_624350.1 |
| Penicillium brasilianum partitivirus 1 (PbPV1) | *Partitiviridae* | AZT88608.1 |
| Penicillium stoloniferum virus S (PsVS) | *Partitiviridae* | YP_052856.2 |
| Rosellinia necatrix partitivirus 1-W8 (RnPV1-W8) | *Partitiviridae* | YP_392480.1 |
| Sclerotinia sclerotiorum partitivirus S (SsPVS) | *Partitiviridae* | YP_003082248.1 |
| Ustilaginoidea virens partitivirus 3 (UvPV3) | *Partitiviridae* | AGJ03719.1 |
| Beauveria bassiana victorivirus 1 (BbVV1) | *Totiviridae* | AMQ11131.1 |
| Coniothyrium minitans RNA virus (CmRV) | *Totiviridae* | ALM62231.1 |
| Magnaporthe oryzae virus 1 (MoV1) | *Totiviridae* | BAD60833.1 |
| Penicillium aurantiogriseum totivirus 1 (PaTV1) | *Totiviridae* | YP_009212848.1 |
| Alternaria dianthicola dsRNA virus 1 (AdRV1) | Proposed family Unirnaviridae | QRZ20363.1 |
| Alternaria longipes dsRNA virus 1 (AlRV1) | Proposed family Unirnaviridae | YP_009052469.1 |
| Aspergillus lentulus non-segmented dsRNA virus 1 (AlNRV1) | Proposed family Unirnaviridae | BCH36647.1 |
| Beauveria bassiana non-segmented RNA virus 1 (BbNRV1) | Proposed family Unirnaviridae | AZT88649.1 |
| Beauveria bassiana RNA virus 1 (BbRV1) | Proposed family Unirnaviridae | AKC57301.1 |
| Colletotrichum gloeosporioides RNA virus 1 (CgRV1) | Proposed family Unirnaviridae | QED88100.1 |
| Colletotrichum higginsianum non-segmented dsRNA virus 1 (ChNRV1) | Proposed family Unirnaviridae | YP_009177217.1 |
| Combu double-strand RNA mycovirus (CdsRV1) | Proposed family Unirnaviridae | QAB47444.1 |
| Erysiphe necator associated non-segmented virus 1 (EnNRV1) | Proposed family Unirnaviridae | QKK35380.1 |
| Fusarium culmorum virus 1 (FcV1) | Proposed family Unirnaviridae | QIC51517.1 |
| Fusarium oxysporum virus 1 (FoV1) | Proposed family Unirnaviridae | WNN27075.1 |
| Penicillium citrinum non-segmented dsRNA virus 1 (PcNRV1) | Proposed family Unirnaviridae | WEY07680.1 |
| Penicillium janczewskii Beauveria bassiana-like virus 1 (PjBlV1) | Proposed family Unirnaviridae | ALO50135.1 |
| Penicillium miczynskii RNA virus 1 (PmRV1) | Proposed family Unirnaviridae | QDB74980.1 |
| Trichoderma harzianum mycovirus 1 (ThV1) | Proposed family Unirnaviridae | AYU71187.1 |
| Ustilaginoidea virens RNA virus M (UvRVM) | Proposed family Unirnaviridae | YP-009094186.1 |
| Ustilaginoidea virens unassigned RNA virus HNND-1  (UvURV-HNND1) | Proposed family Unirnaviridae | YP_009154709.1 |
| Ustilaginoidea virens unassigned RNA virus HNND-1-A  (UvURV-HNND1A) | Proposed family Unirnaviridae | UVX28908.1 |
| Conidiobolus non-segmented RNA virus 1 (CNRV1) | Proposed genus Ustivirus | QKL20127.1 |
| Nigrospora oryzae unassigned RNA virus 1 (NoURV1) | Proposed genus Ustivirus | ALR87111.1 |
| Phytophthora cactorum usti-like virus 1 (PcUlV1) | Proposed genus Ustivirus | QUA12647.1 |
| Purpureocillium lilacinum nonsegmented virus 1 (PlNV1) | Proposed genus Ustivirus | AOO52902.1 |
| Ustilaginoidea virens nonsegmented virus 1 (UvNV1) | Proposed genus Ustivirus | AIE77248.1 |
| Ustilaginoidea virens nonsegmented virus 2 (UvNV2) | Proposed genus Ustivirus | YP_009553682.1 |
