# Supplementary material for: A Novel Strain of Fusarium oxysporum Virus 1 Isolated from Fusarium oxysporum f. sp. niveum Strain X-GS16 Influences Phenotypes of F. oxysporum Strain HB-TS-YT-1hyg
Source: J Fungi (Basel). 2024 Mar 27;10(4):252. doi: 10.3390/jof10040252 (PMC11050907; doi:10.3390/jof10040252)
Supplement: Supplementary file 1 [file jof-10-00252-s001.zip › Table S3.docx]

**Table S3.** The information of 18 representative members in the proposed family Unirnaviridae retrieved from GenBank database (National Center for the Biotechnology Information) and used to analyze the hypothetical -1 frameshifting motifs.

| **Reference Virus** | **Family** | **Hypothetical -1 Frameshifting Motif** | **GenBank Accession Number** | |
| --- | --- | --- | --- | --- |
| Alternaria dianthicola dsRNA virus 1 (AdRV1) | Proposed family Unirnaviridae | G GAU UUU UAG | | MT241326.1 |
| Alternaria longipes dsRNA virus 1 (AlRV1) | Proposed family Unirnaviridae | G GAU UUU UAG | | NC_024703.1 |
| Aspergillus lentulus non-segmented dsRNA virus 1 (AlNRV1) | Proposed family Unirnaviridae | G GAU UUU UAG | | BCH36647.1 |
| Beauveria bassiana non-segmented RNA virus 1 (BbNRV1) | Proposed family Unirnaviridae | G GAU UUU UAG | | MK279499.1 |
| Beauveria bassiana RNA virus 1 (BbRV1) | Proposed family Unirnaviridae | G GAU UUU UAG | | KM233415.1 |
| Colletotrichum gloeosporioides RNA virus 1 (CgRV1) | Proposed family Unirnaviridae | G GAU UUU UAG | | MK926570.1 |
| Colletotrichum higginsianum non-segmented dsRNA virus 1 (ChNRV1) | Proposed family Unirnaviridae | G GAU UUU UAG | | NC_028242.1 |
| Combu double-strand RNA mycovirus (CdsRV1) | Proposed family Unirnaviridae | G GAU UUU UAG | | MH990637.1 |
| Erysiphe necator associated non-segmented virus 1 (EnNRV1) | Proposed family Unirnaviridae | G GAU UUU UAG | | MN617774.1 |
| Fusarium culmorum virus 1 (FcV1) | Proposed family Unirnaviridae | G GAU UUU UAA | | MN187541.1 |
| Fusarium oxysporum virus 1 (FoV1) | Proposed family Unirnaviridae | G GAU UUU UAA | | OR372790.1 |
| Penicillium citrinum non-segmented dsRNA virus 1 (PcNRV1) | Proposed family Unirnaviridae | G GAU UUA AAU UUC UAG | | OP103962.1 |
| Penicillium janczewskii Beauveria bassiana-like virus 1 (PjBlV1) | Proposed family Unirnaviridae | G GAU UUC UAG | | KT601106.1 |
| Penicillium miczynskii RNA virus 1 (PmRV1) | Proposed family Unirnaviridae | U UUA AAC UUU UAG | | MK584820.1 |
| Trichoderma harzianum mycovirus 1 (ThV1) | Proposed family Unirnaviridae | G GAU UUU AUG UAA | | MH155602.1 |
| Ustilaginoidea virens RNA virus M (UvRVM) | Proposed family Unirnaviridae | G GAU UUU UAA | | NC_025367.1 |
| Ustilaginoidea virens unassigned RNA virus HNND-1 (UvURV-HNND1) | Proposed family Unirnaviridae | U UUA AAC UUC UAG | | NC_027427.1 |
| Ustilaginoidea virens unassigned RNA virus HNND-1-A (UvURV-HNND1A) | Proposed family Unirnaviridae | G GAU UUA AAC UUC UAG | | ON791651.1 |

Note: The nucleotides, G GAU UUU, G GAU UUA, G GAU UUC, or U UUA AAC, were the hypothetical -1 frameshifting motifs, and UAG or UAA were the stop codon.
